# Lung hypoplasia in newborn rabbits with a diaphragmatic hernia affects pulmonary ventilation but not perfusion

**DOI:** 10.1038/pr.2017.91

**Published:** 2017-05-31

**Authors:** Andreas W Flemmer, Marta Thio, Megan J Wallace, Katie Lee, Marcus J Kitchen, Lauren Kerr, Charles C Roehr, Andreas Fouras, Richard Carnibella, Jaccques C Jani, Philip DeKoninck, Arjan B te Pas, James T Pearson, Stuart B Hooper

**Affiliations:** 1 Division of Neonatology University Children’s Hospital and Perinatal Center, Ludwig Maximilian University, Munich, Germany; 2 Newborn Research Centre, The Royal Women’s Hospital, Melbourne, Australia; 3 Department of Obstetrics and Gynaecology, The University of Melbourne, Melbourne, Australia; 4 Ritchie Centre, Hudson Institute of Medical Research, Melbourne, Australia; 5 Department of Obstetrics and Gynaecology, Monash University, Melbourne, Australia; 6 School of Physics and Astronomy, Monash University, Melbourne, Australia; 7 Department of Mechanical and Aerospace Engineering, Monash University, Melbourne, Australia; 8 Department of Obstetrics and Gynecology, University Hospital Brugmann, Université Libre de Bruxelles, Brussels, Belgium; 9 Division of Neonatology, Department of Pediatrics, Leiden University Medical Center, Leiden, The Netherlands; 10 Monash Biomedical Imaging Facility, Department of Physiology, Monash University, Melbourne, Australia; 11 Australian Synchrotron, Melbourne, Australia; 12 National Cerebral and Cardiovascular Center, Suita, Japan

## Abstract

**Background:**

A congenital diaphragmatic hernia (DH) can result in severe lung hypoplasia that increases the risk of morbidity and mortality after birth; however, little is known about the cardiorespiratory transition at birth.

**Methods:**

Using phase-contrast X-ray imaging and angiography, we examined the cardiorespiratory transition at birth in rabbit kittens with DHs. Surgery was performed on pregnant New Zealand white rabbits (*n*=18) at 25 days’ gestation to induce a left-sided DH. Kittens were delivered at 30 days’ gestation, intubated, and ventilated to achieve a tidal volume (*V*_t_) of 8 ml/kg in control and 4 ml/kg in DH kittens while they were imaged.

**Results:**

Functional residual capacity (FRC) recruitment and *V*_t_ in the hypoplastic left lung were markedly reduced, resulting in a disproportionate distribution of FRC into the right lung. Following lung aeration, relative pulmonary blood flow (PBF) increased equally in both lungs, and the increase in pulmonary venous return was similar in both control and DH kittens.

**Conclusion:**

These findings indicate that nonuniform lung hypoplasia caused by DH alters the distribution of ventilation away from hypoplastic and into normally grown lung regions. During transition, the increase in PBF and pulmonary venous return, which is vital for maintaining cardiac output, is not affected by lung hypoplasia.

A congenital diaphragmatic hernia (CDH) is a common congenital abnormality that is characterized by an incomplete formation of the diaphragm, which fails to separate the abdominal and thoracic compartments during fetal development (1, 2, 3). Abdominal organs enter the chest causing lung hypoplasia by limiting the intrathoracic space available for lung growth. CDHs most commonly occur on the left side (85–90%), but can occur on the right (~10%) or on both sides (~2%) as well, and, when it is the only defect, survival rates are 60–70% depending on the severity of lung hypoplasia (4).

As the lungs are not required for gas exchange before birth, CDH infants can be healthy *in utero*, but at birth over 90% suffer severe respiratory insufficiency (1, 2, 3), resulting in high mortality and morbidity rates (1). As the reduction in lung growth is not uniform, marked functional differences exist across the lungs, making it difficult to ventilate these infants without injuring the lung (5). As little is known about the fetal-to-neonatal transition in infants with CDH, the current care guidelines are largely based upon expert opinion (1, 3).

The lungs of CDH infants also have an abnormally developed vascular bed with a small cross-sectional area (6, 7). As a large cross-sectional area is required to achieve a low resistance, a reduced cross-sectional area will limit the ability of the lungs to reduce vascular resistance after birth. This not only contributes to persistent pulmonary hypertension of the newborn, but also reduces pulmonary venous return and preload for the left ventricle and maintains high afterload on the right ventricle (8, 9). Thus, both left and right ventricular outputs remain dependent upon persisting shunts (foramen ovale and ductus arteriosus). As a result, pulmonary hypertension of the newborn, persistence of fetal vascular shunts, and low cardiac outputs are common in CDH infants (6); however, there is little information on how these infants transition at birth.

At birth, the transition from fetal to newborn life commences with lung aeration and the onset of pulmonary ventilation. This is achieved through the clearance of airway liquid into the surrounding lung tissue, which allows the entry of air and the creation of a functional residual capacity (FRC) within three to five breaths. This process stimulates a large increase in the pulmonary blood flow (PBF), which enhances pulmonary gas exchange and restores the reduction in ventricular preload lost following umbilical cord clamping. As a result, the transition to newborn life involves a complex sequence of cardiorespiratory changes that can occur within minutes of birth.

Our aim was to use phase-contrast X-ray (PCX) imaging and angiography to measure the temporal and spatial patterns of lung aeration, the distribution of ventilation, and the increase in PBF at birth in mechanically ventilated newborn rabbits with a left-sided diaphragmatic hernia (DH). We hypothesized that unilateral pulmonary hypoplasia will markedly alter the uniformity of lung aeration and the distribution of ventilation, and produce a smaller and nonuniform increase in PBF.

## Methods

### Animal Procedures

Animal procedures were approved by the Monash University Animal Ethics Committee and the SPring-8 Animal Care and Use Committee. Experiments were conducted at the SPring-8 synchrotron, Japan, as previously described (10, 11, 12). Pregnant New Zealand white rabbits (*n*=18) underwent aseptic surgery at 25 days’ gestation to induce a left-sided DH in two fetuses per rabbit as previously described (13, 14).

At 30 days’ gestation (near-term), rabbit anesthesia was induced (8 mg kg^−1^ intravenous; Propofol, Schering-Plough Animal Health, Tokyo, Japan) and maintained with isoflurane (1–4% Isoflurane, Dainippon Sumitomo Pharma, Osaka, Japan). Rabbit kittens were exteriorized, sedated with sodium pentobarbitone (13 mg kg^−1^ intraperitoneal; Somnopentyl, Kyoritsu Seiyaku, Tokyo, Japan), and an endotracheal tube (18G; Becton Dickinson, Franklin Lakes, NJ) was inserted. The umbilical cord was ligated, and kittens were delivered and positioned in a plethysmograph or Perspex holder (for angiography) for imaging. The endotracheal tube was connected to a custom-built ventilator (15) and kittens were imaged either using (i) PCX imaging or (ii) PCX combined with angiography; these kittens also had a jugular vein catheter (24G Intracath; Becton Dickinson) inserted non-occlusively (10, 11).

All kittens were ventilated with air for ~10 min, using a peak inflation pressure commencing at 25 cmH_2_O and a positive end-expiratory pressure of 5 cmH_2_O at 60 breaths per min. The peak inflation pressure was changed to achieve a tidal volume of 4–5 ml/kg in DH kittens and 7–8 ml/kg in control kittens, as measured using a pneumotach. All animals were killed at the conclusion of the experiment with an overdose of sodium pentobarbitone (intraperitoneal Somnopentyl, Kyoritsu Seiyaku). Following death, kittens were imaged using computed tomography and three-dimensional reconstructions of the lungs. The lungs were removed, weighed, and pressure-fixed at 20 cmH_2_O in 4% paraformaldehyde via the airways before they were embedded in paraffin, sectioned at 5 μm, and stained with hematoxylin and eosin, as previously described (16).

### X-ray Image Acquisition

#### PCX and angiographic imaging

PCX images were obtained using a synchrotron source tuned to 24 keV (refs. 11, 12, 17) or to 33.2 keV for angiographic imaging (10, 11). A Hamamatsu ORCA flash C11440-22C detector (PCX imaging) or a scientific-CMOS detector (pco.edge; PCO AG, Kehlheim, Germany; angiographic imaging) and a tandem lens system were used; pixel size was 15.3 μm and the field of view was 29(*W*) × 30(*H*)mm^2^. Images were acquired at 10 Hz for PCX imaging and 20 Hz for angiography. Iodine boluses (Iomeron 350 mg ml^−1^; Bracco-Eisai, Tokyo, Japan; 1.5 μl g^−1^ body weight at 11 ml s^−1^) were injected via the jugular vein using a remote-controlled pump (PHD2000, Harvard Apparatus, Holliston, MA) before ventilation and at three different time points during and after lung aeration.

### Image Processing and Analysis

#### PCX and angiographic imaging

Total and regional lung gas volumes were measured from images as previously described (18). The chest was divided into quadrants by partitioning each frame into left and right lungs above and below the seventh rib, to measure gas volumes within the upper and lower sections of the left and right lungs. Measures of FRC and peak inflation and tidal volumes within each region were made throughout the experimental period.

To analyze angiographic images, regions of interest (ROIs) were drawn over the vessels, and the mean intensity within that region was recorded for every frame across all boluses (10, 11). All changes in the mean intensity were then converted to percentage change from background, which was the average of the ROI over five iodine-free frames.

To quantify pulmonary venous return and cardiac shunting through the foramen ovale, the mean intensity profile for a ROI drawn over the left ventricle was recorded for two separate time periods: (i) The first was immediately after iodine had entered the right atrium until it entered the PAs. During this time, any iodine entering the left atrium and ventricle must have travelled through the foramen ovale (Figure 2e). (ii) The second was after iodine had dispersed from the PAs and the lungs (seen as transient uniform darkening of the lung tissue). At this time, any iodine entering the left ventricle is derived from the pulmonary venous return.

To measure pulmonary arterial transit time and flow, ROIs were drawn over the main pulmonary artery and the left and right PAs just outside of the heart shadow. The profile of the mean intensity change (for the ROI) was measured across all frames, and the iodine entry and exit times were defined as the time when the mean increased and then decreased to the half-peak opacification, respectively (10, 11). The transit time was defined as the time taken to reach half-peak opacification in the left and right PAs after half-peak opacification was measured in the main pulmonary artery. Relative flow was then approximated by dividing the peak intensity by the time between iodine entry and exit (10).

### Statistical Analysis

Lung volume (FRC, *V*_t_ and peak inflation volumes) measurements, changes in the distribution of iodine, pulmonary arterial transit time, and pulmonary arterial flow were analyzed using a two-way repeated measures analysis of variance followed by a Holm–Sidak *post hoc* analysis. Differences in kitten weights and whole-lung volumes were assessed using a Student’s *t*-test. *P*<0.05 was considered statistically significant. Data are expressed as mean±SEM.

## Results

### Animal and Anatomical Data

The success rate for DH fetuses with abdominal contents in the chest at delivery was 55.6%. Two DH kittens, but no control kittens, developed a pneumothorax and were excluded from the analysis. The mean body weights of control kittens (32.6±1.2 g; *n*=10) were similar to those of DH kittens (34.1±1.0 g; *n*=9). Whole-lung volumes were significantly reduced in DH kittens (51±3 μl/g) compared with those in controls (67±2 μl per g body weight, *P*<0.05). The degree of hypoplasia varied between DH kittens, as indicated by a higher variation in lung volume-to-body weight ratios in DH kittens (20.3%) than in control kittens (12.2%).

**Figure 1:**
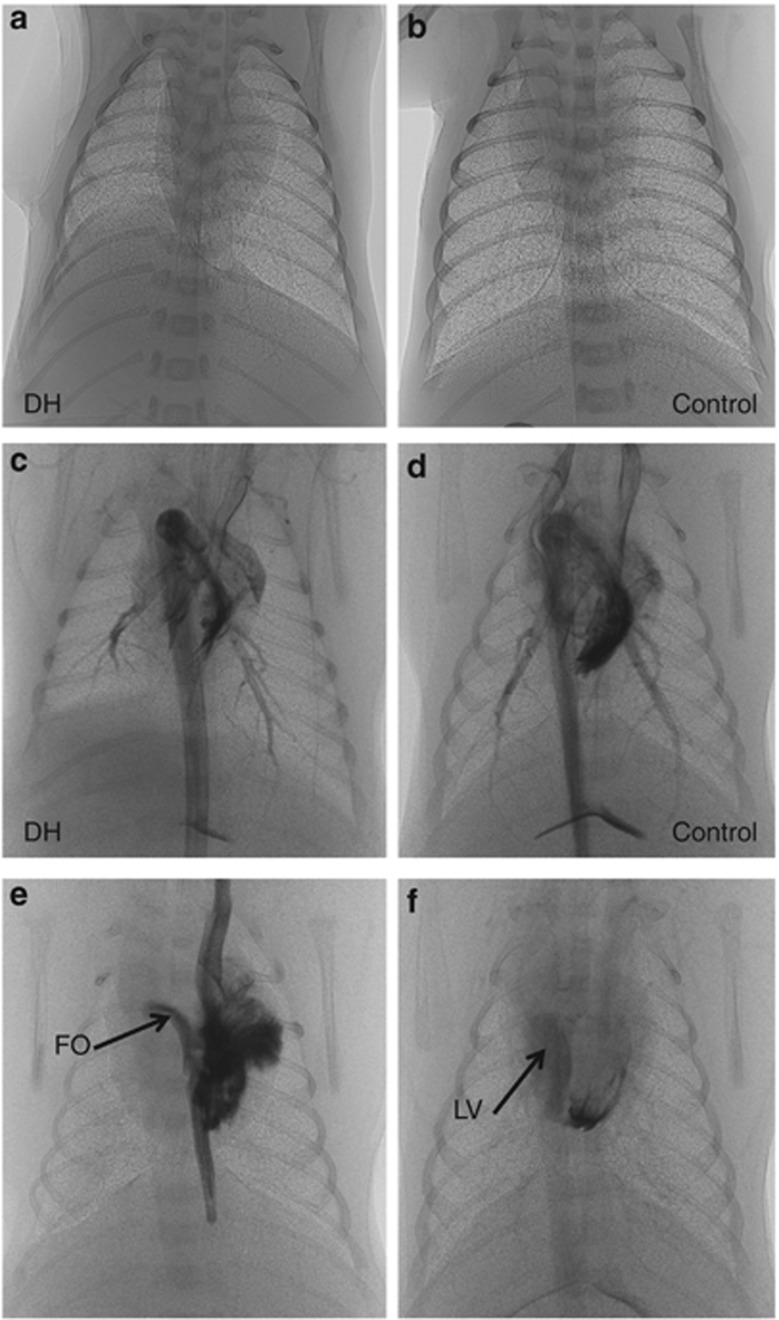
Phase-contrast X-ray (PCX) images of (**a**) a kitten with a left-sided diaphragmatic hernia (DH); (**b**) control kitten; (**c**) a kitten with a left-sided DH using angiography to show the pulmonary arterial tree; (**d**) a control kitten using angiography to show the pulmonary arterial tree; (**e**) a DH kitten using angiography to image the foramen ovale (FO); and (**f**) a control kitten using angiography to image the left ventricle (LV) and quantify pulmonary venous return.

**Figure 2:**
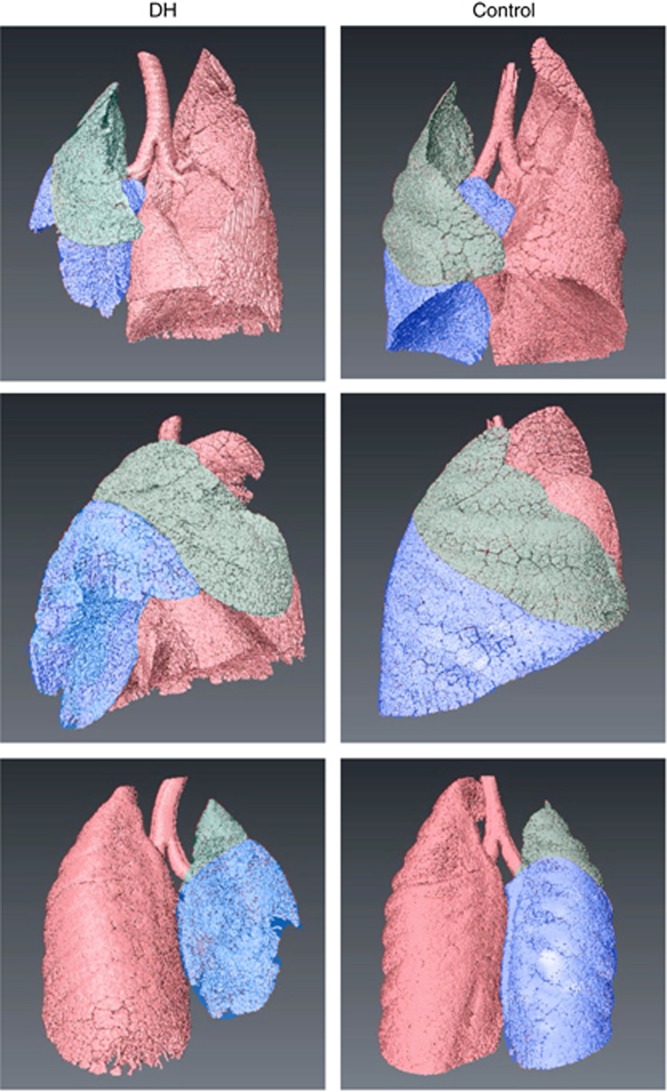
Computed tomographic three-dimensional reconstruction of lungs from a diaphragmatic hernia (DH) kitten and a control kitten. In DH kittens, the right lung (light red) appeared unaffected, whereas the left upper lobe (green) was slightly reduced in size and the left lower lobe (blue) was markedly reduced in size.

PCX images show the extent of the hypoplasia (mostly limited to the left side) and the variability between kittens (Figure 1). Computed tomography analysis revealed that reduced lung development in DH kittens was mostly limited to the left lower lobe, although the left upper lobe was also affected to a lesser degree (Figure 2). Histologically, the left lower lobes in DH kittens appeared immature in structure, with thick inter-airway walls and simplified distal airways, compared with controls (data not shown).

### Temporal and Spatial Pattern of Lung Aeration

#### FRC recruitment

**Figure 3:**
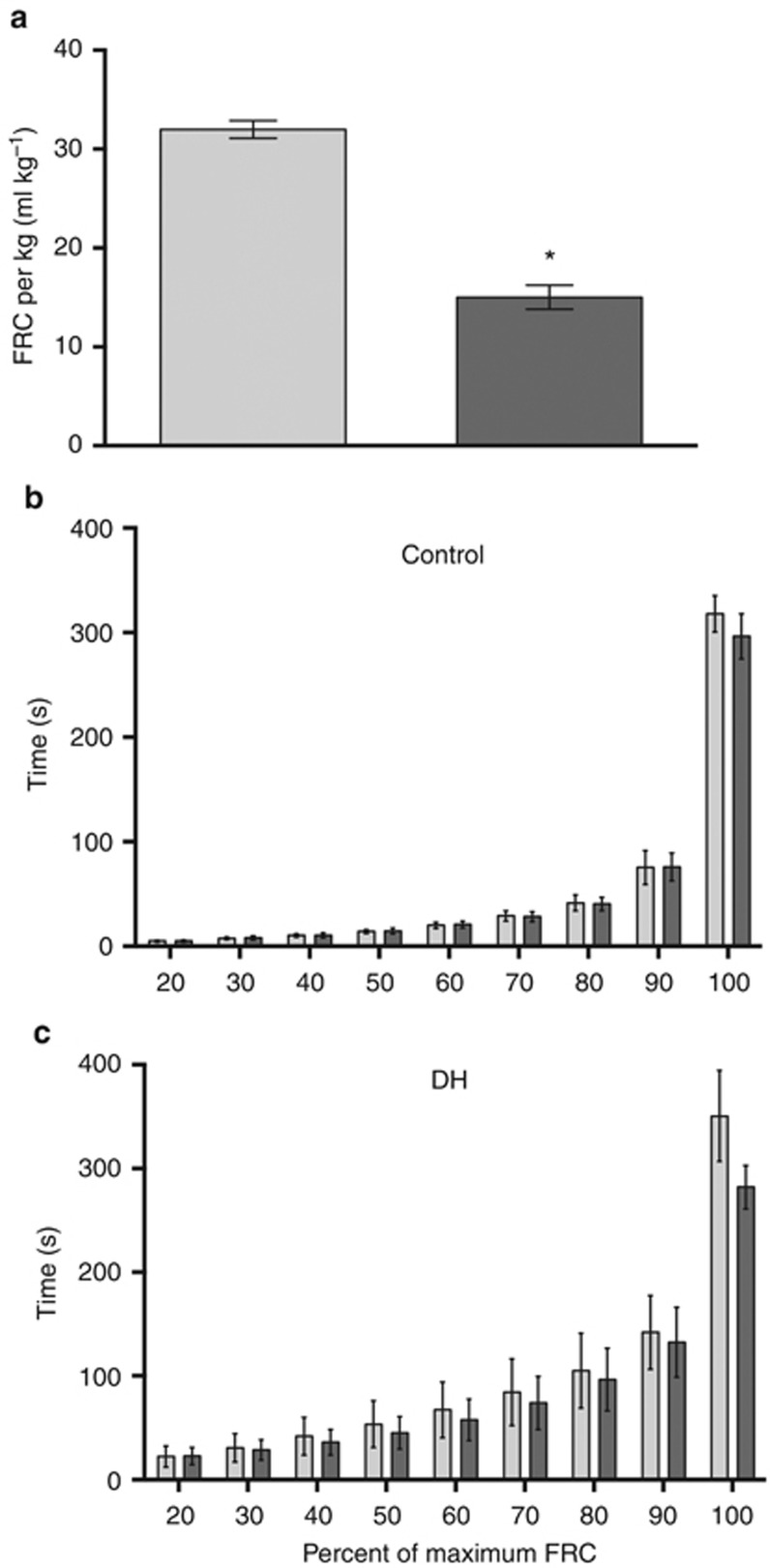
Measures of total functional residual capacity (FRC; **a**) and the temporal pattern of FRC recruitment in the left (hypoplastic; dark gray) and right (light gray) lungs of control (**b**) and diaphragmatic hernia (DH; **c**) kittens. The temporal pattern of FRC recruitment is displayed as the time taken to reach 20–100% of the measured FRC for each lung (mean±SEM, *P<0.05).

Following lung aeration, the maximum FRC achieved was significantly less in DH kittens than in control kittens (Figure 3a). In DH kittens, FRC in the ipsilateral left lung was reduced compared with that in the contralateral right lung, resulting in a marked difference in FRC distribution between the left and right lungs. Although the FRC was also greater in the right lung of control kittens, this difference was expected because of the size difference between the two lungs. Nevertheless, the percentage of total FRC present in the left lung was significantly (*P*=0.002) less in DH kittens than in control kittens, whereas the percentage of total FRC in the right lung was significantly greater in DH kittens than in control kittens (*P*=0.002). In DH kittens, the reduced FRC in the left lung was accounted for by a reduction in FRC in the lower lobe, as the FRC in the upper left lobe was similar in both DH and control kittens.

In the hypoplastic left lung of DH kittens, the temporal pattern of FRC recruitment was similar to that of the contralateral right lung and both the left and right lungs of control kittens (Figure 3b). Expressed as a percentage of the maximum FRC, the hypoplastic left lung of DH kittens tended to take longer than the contralateral right lung to achieve each FRC percentile (20–100% of FRC), despite achieving lower volumes. The left lung took 350±44 s to reach 100% FRC, whereas the right lung took 282±21 s (Figure 3c).

#### Tidal volumes

**Figure 4:**
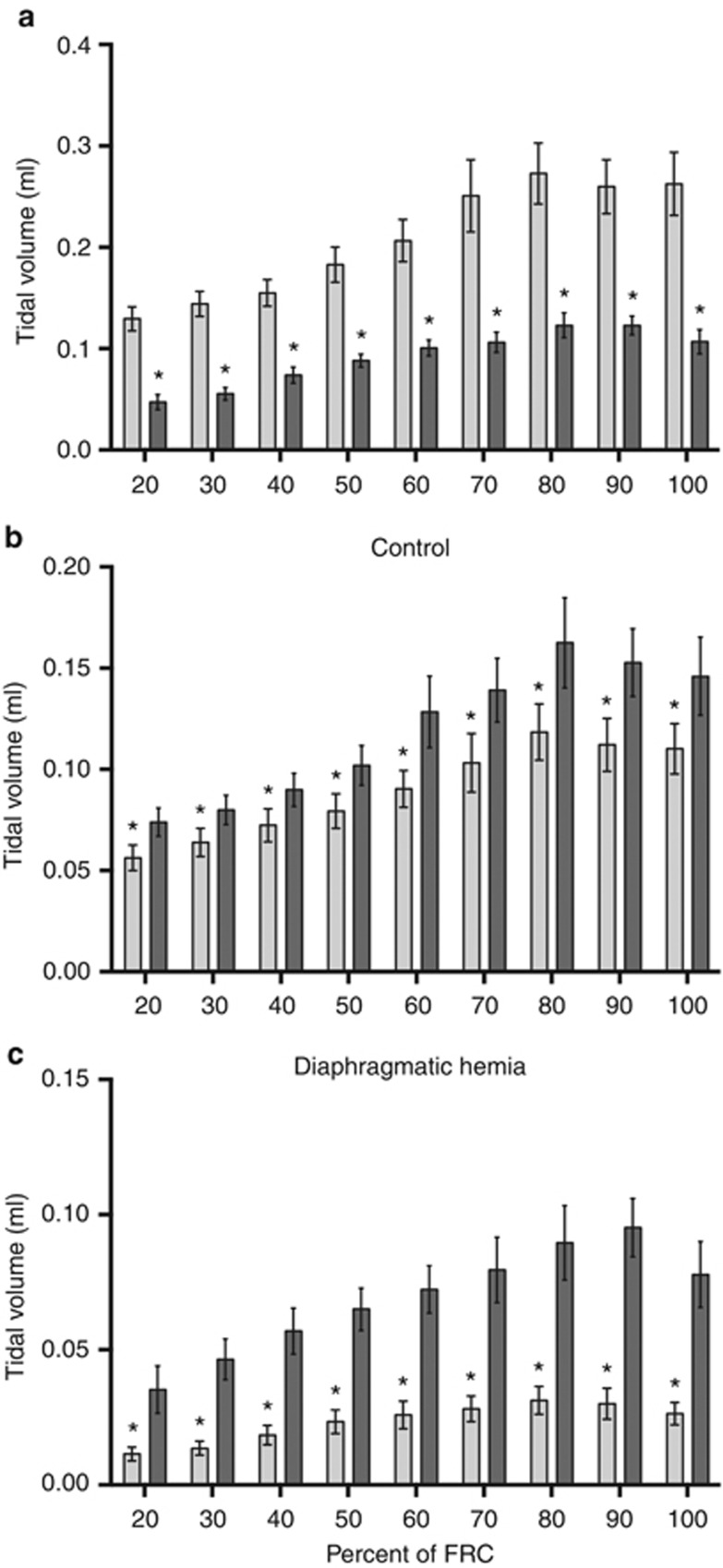
The tidal volume (**a**) in control (light gray) and diaphragmatic hernia (DH; dark gray) lungs and the distribution of tidal volume between the left (hypoplastic; light gray) and right (dark gray) lungs of control (**b**) and DH (**c**) kittens (mean±SEM, *P<0.05).

**Figure 5:**
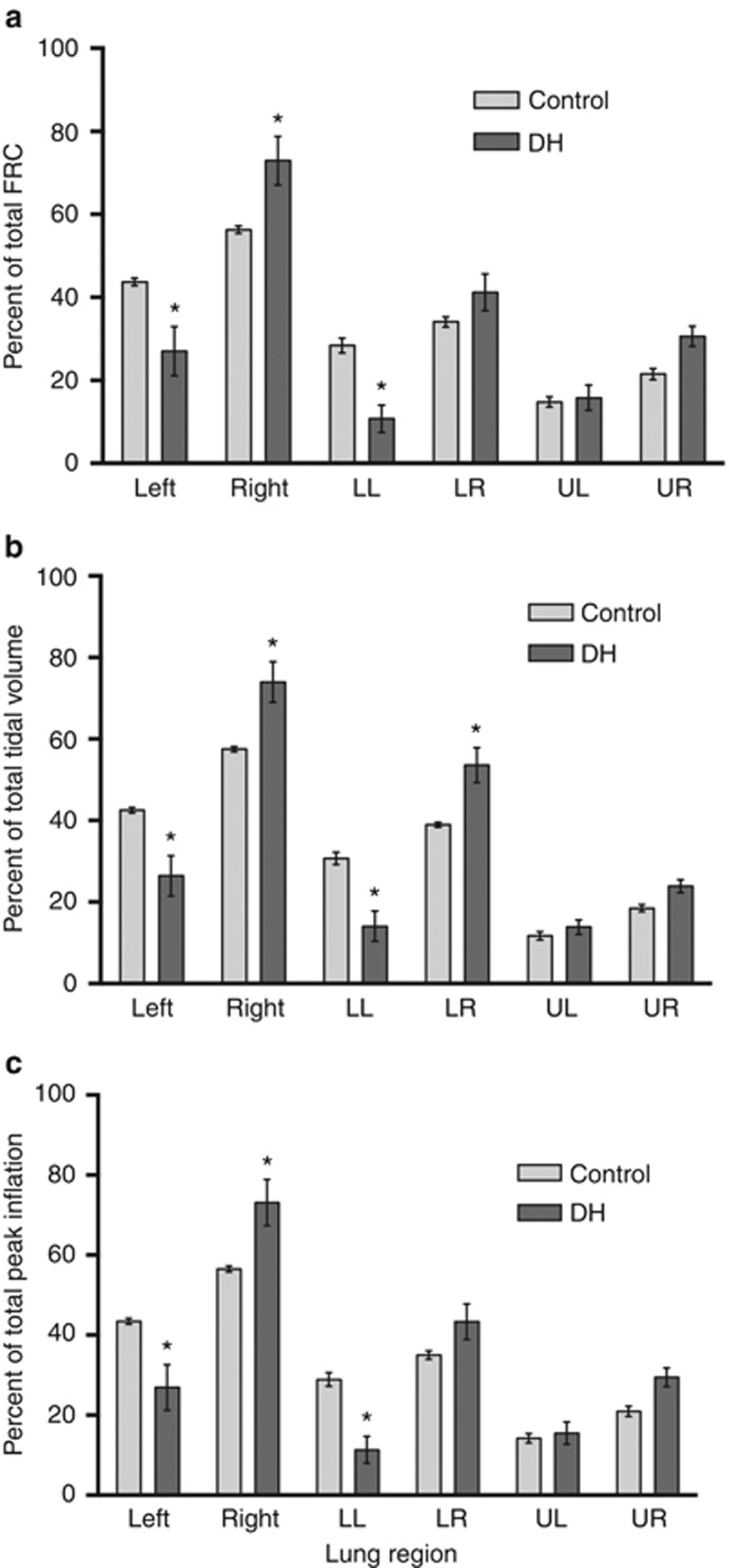
The proportional distribution of functional residual capacity (FRC; **a**), incoming tidal volume (**b**), and peak inflation volumes (**c**) in kittens with a diaphragmatic hernia (DH; dark gray) and in control kittens (light gray; mean±SEM, *P<0.05). LL, lower left lung; LR, lower right lung; UL, upper left lung; UR, upper right lung.

To reduce the risk of a pneumothorax, we aimed to deliver a smaller tidal volume in DH kittens than in control kittens. The mean *V*_t_ in DH kittens at each stage of lung aeration was significantly lower than that in control kittens (Figure 4a), but it tended (*P*=0.08) to require a higher peak inflation pressure (19.6±0.9 vs. 17.6±0.7 cmH_2_O) to achieve this *V*_t_. Thus, dynamic lung compliance was less in DH kittens (0.51±0.02 ml/kg per cmH_2_O) compared with control kittens (0.68±0.04 ml/kg per cmH_2_O; *P*=0.0017). We observed a highly significant (*P*=0.0035) relationship between lung volume-to-body weight ratios and dynamic lung compliance, indicating that low lung compliance is closely related to LH. In DH kittens, the percentage of *V*_t_ entering the left lung was reduced, whereas the percentage of *V*_t_ entering the right lung was increased, compared with controls (Figure 5b).

During lung aeration, the temporal pattern of tidal ventilation recruitment in the left and right lungs was similar in both DH and control kittens, whereas the magnitude of the distribution between the two lungs was very different between the two groups (Figure 4b). In DH kittens, at 20% of maximum FRC, only 27.0±6.4% of the *V*_t_ entered the hypoplastic left lung, whereas 73.0±6.4% entered the right lung. In contrast, 43.1±1.8% of the tidal volume entered the left lung and 56.9±1.8% entered the right lung in control kittens. When FRC was at 100%, the difference in *V*_t_ distribution between the left and right lungs in both DH (26.4±4.5% in left and 73.6±4.5% in right) and control (43.3±0.7% in left and 56.7±0.7% in right) kittens remained similar to that measured at 20% (Figure 4b).

#### End-inflation lung volumes

Following aeration, lung volumes at peak inflation were reduced in DH kittens compared with those in control kittens, largely due to reduced volumes in the hypoplastic left lung (Figure 5c). At peak inflation, the proportion of air in the hypoplastic left lung was less than that in the contralateral right lung and in the left lung of control kittens due to a reduction in the left lower lobe (Figure 5c). In contrast, the proportion of air in the contralateral right lung of DH kittens at peak inflation was greater than that in the right lung of control kittens, due to an increase in both right upper and lower lobes.

### Pulmonary Arterial Blood Flow Changes

#### Pulmonary venous return and flow through the foramen ovale

**Figure 6:**
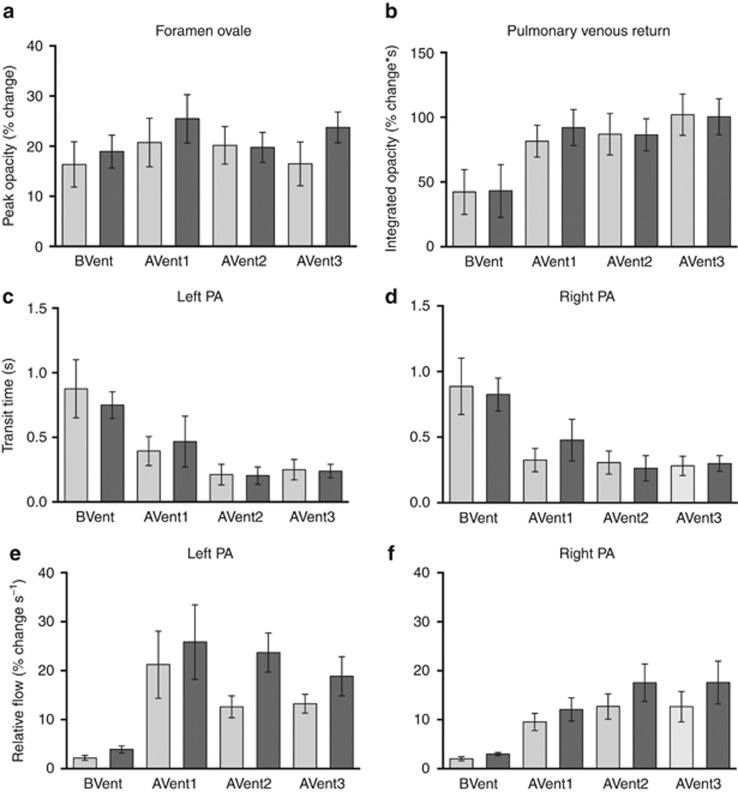
Relative measures of (**a**) right-to-left blood flow through the foramen ovale, (**b**) pulmonary venous return and blood flow in the left (**e**) and right (**f**) pulmonary arteries (PAs), as well as measures of blood flow transit times in the left (**c**) and right (**d**) pulmonary arteries (PAs)s in diaphragmatic hernia (DH; dark gray) and control kittens (light gray). BVent indicates measurements prior to ventilation, AVent1-3 indicate measurements after the first transitional breaths.

Relative measures of pulmonary venous return increased in response to lung aeration, but this increase was similar in both control and DH kittens. Similarly, no differences in relative right-to-left flow through the foramen ovale were detected between both control and DH kittens, although the flow tended to be higher in DH kittens, particularly after lung aeration (Figure 6a).

#### Pulmonary arterial transit time and flow

The pulmonary artery (PA) transit times for iodine were significantly decreased from 0.82±0.22 and 0.89±0.21 s in the left and right PAs, respectively, to 0.25±0.08 and 0.31±0.09 s in control kittens following lung aeration (Figure 6c). Similarly, in DH kittens, lung aeration reduced PA transit times from 0.75±0.10 and 0.83±0.13 s to 0.24±0.05 and 0.30±0.06 s in the left and right lungs, respectively. No differences in transit times within the left and right PAs were observed between both DH and control kittens either before or after lung aeration (Figure 6).

Following lung aeration, relative measures of PBF were increased from 2.2±0.5 and 2.0±0.4%.s^−1^ to 21.2±6.8 and 9.5±1.7%.s^−1^ in the left and right PAs, respectively, in control kittens. Similarly, in DH kittens, relative PBF increased from 3.9±0.7 and 3.0±0.3%.s^−1^ to 25.8±7.6 and 12.1±2.4%.s^−1^ in the left and right PAs, respectively. No differences in relative PBF were observed between both control and DH kittens or left and right lungs either before or after lung aeration (Figure 6e).

## Discussion

Although much research has focused on the developmental consequences of CDH, no studies have investigated how lung hypoplasia affects the cardiorespiratory transition at birth. We found that a left-sided DH, which causes hypoplasia of the lung’s left lower lobe, alters the spatial pattern of lung aeration and the distribution of ventilation between the left and right lungs. The hypoplastic left lung had a much lower FRC, mostly because of reduced volumes within the left lower lobe, and total lung compliance was closely related to lung size. As a result, the proportion of FRC within the right lung of DH kittens was greater than that in control kittens, although the absolute volumes were similar. *V*_t_ distribution was also markedly altered in DH kittens throughout the period of lung aeration, with most air entering the right lung. We also found that lung aeration increased the relative PBF and pulmonary venous return and reduced the PA transit times to a similar extent in both left (hypoplastic) and right (unaffected) lungs of DH kittens as well as in the left and right lungs of control kittens. This unexpected finding potentially indicates that the initial vasodilation induced by lung aeration can over-ride structural differences in the pulmonary vascular bed. However, we cannot rule out the possibility that an increased vasoreactivity may have occurred if ventilation had continued for a longer period of time after birth, resulting in the gradual development of pulmonary hypertension.

### Lung Aeration and Pulmonary Ventilation

After birth, airway liquid clearance mostly occurs because of hydrostatic pressures generated during inspiration (or positive pressure inflation), which promotes liquid movement from the airways into perialveolar tissue (19, 20). The resistance to moving fluid through the airways depends upon the size and total cross-sectional area of the airways and the viscosity of the fluid, whereas the movement of liquid across the distal airway wall is largely influenced by surface area (20). Thus, the resistance and time taken to clear airway liquid increase in preterm kittens, likely due to the combined effect of smaller airway diameters and reduced distal airway surface areas (21). We have previously shown that in mechanically ventilated kittens of a similar gestational age, lung aeration occurs very rapidly, resulting in full tidal volume and FRC recruitment within 3–5 min.

It is surprising that the temporal pattern of FRC recruitment was similar in the left and right lungs of DH kittens, as we expected the cross-sectional area of the airways and surface area to be reduced in the hypoplastic left lung. However, as the FRCs were lower in the hypoplastic left lung, the liquid clearance rates were proportional to lung size, indicating that the liquid volume cleared per unit time was reduced in the left lung, possibly because of the simplified distal airway structure. Our computed tomography analysis indicated that the predominant effect of the DH in our model was a reduction in the left lower lobe size (Figure 2), which is consistent with the finding that reduced FRC levels in DH kittens were due to a reduction within the lower lobe of the left lung. Although the absolute FRC volume of the right lung was similar in DH kittens and controls, expressed as a proportion of total FRC, the FRC in the right lung was proportionately higher than that in controls.

The proportion of the incoming *V*_t_ entering the left hypoplastic lung and the peak inflation achieved were significantly reduced compared with both the right lung of DH kittens and the left lung of control kittens. As DH kittens were expected to have a smaller lung, we ventilated them with a lower *V*_t_ (4 ml/kg) to reduce the risk of a pneumothorax. In control kittens, ~43% of this *V*_t_ entered the left lung and 57% entered the right lung, which reflects the known size difference between the left and right lungs (Figure 4b). However, in DH kittens, only 25% of the *V*_t_ entered the left lung and 75% entered the right lung (Figure 4c). On the basis of this distribution, we calculate that if we had used the same *V*_t_ in DH kittens as in control kittens (8 ml/kg), the *V*_t_ entering the right lung would have been ~25% higher in DH kittens (0.19±0.01 ml) than in control kittens (0.14±0.02 ml). Thus, the marked compliance differences between the hypoplastic left lung and the more normal right lung cause a greater shift of the *V*_t_ into the right lung than would be expected due to size differences between the two. This justifies the use of a lower *V*_t_ in CDH infants at birth in order to avoid overventilation and injury in less hypoplastic lung regions (3). However, in our study, the *V*_t_ of the right lung was less in DH kittens (0.10±0.01 ml) than in control kittens (0.15±0.02 ml), indicating that we likely underventilated DH kittens and should have ventilated them at 5–6 ml/kg, rather than at ~4 ml/kg.

### Cardiovascular Parameters

Lung aeration increased relative PBF in both the left and right PAs in both control and DH kittens. This unexpected finding indicates that, at least initially, the increase in PBF induced by lung aeration is not reduced in the hypoplastic lung. Although it is unclear as to how long the hypoplastic lung is able to sustain this high degree of PBF, this may explain why some CDH infants appear to have a “honeymoon” period before they develop pulmonary hypertension. Our previous studies have demonstrated that the increase in PBF induced by partial lung aeration is similar in aerated and unaerated regions (10, 11), resulting in a global increase in PBF that is enhanced but not dependent on increased oxygenation (10). Combined with the findings of this study, it appears that the mechanism responsible for the increase in PBF at birth can over-ride the higher resistance associated with an underdeveloped pulmonary vascular bed. Whether or not this provides a greater stimulus for the pulmonary vascular bed to remodel after birth, or whether the pulmonary vascular bed is hyper-reactive, is currently unclear.

As we expected PBF to be reduced in DH kittens, we also expected that pulmonary venous return and left ventricular preload would be reduced and that right-to-left flow through the foramen ovale would increase. However, we found that pulmonary venous return increased equally in control and DH kittens, which is consistent with the finding that PBF increased similarly in both groups. As iodine was injected into the superior vena cava, only right-to-left flow through the foramen ovale was measured and was derived from the upper body. Before birth, much of the flow through the foramen ovale is derived from umbilical venous return that flows via the ductus venosus and inferior vena cava (22). However, with the loss of this flow following umbilical cord clamping, it is unclear whether flow from the superior vena cava increases to sustain right-to-left flow through the foramen ovale and provide some left ventricular preload before PBF increases (22). Our results indicate that a significant proportion of superior vena cava flow passes through the foramen ovale following cord clamping. We expected this flow to remain high in DH kittens because we expected that if PBF did not increase, then left ventricle preload would depend on sustained or increased right-to-left flow through the foramen ovale. As PBF and pulmonary venous return were similar between groups, clearly left ventricular output was not increasingly reliant on right-to-left flow through the foramen ovale in DH kittens.

A limitation of our study was creation of the DH on day 25 of gestation, which likely reduced the resulting degree of lung hypoplasia. In the rabbit model, usually the DH is created at 23 days of gestation in order to affect lung development during the pseudoglandular phase of lung development. At 25 days, the fetal rabbit lung is approaching the canalicular phase, and, as such, the effect on lung development will more closely reflect late lung hypoplasia. Nevertheless, we demonstrated significant asymmetric differences in lung function between the ipsilateral and contralateral lungs as well as between control and DH lungs, indicating that the mild changes in lung development produce profound changes in function.

In summary, we found that a DH affects the cardiorespiratory transition at birth. The distribution of ventilation was directed away from hypoplastic lung regions into other regions to a greater degree than is expected based on lobe-size differences. This is likely due to an immature lung structure that reduced lung compliance to a greater degree than that expected because of lung size. In contrast, the increases in PBF were not affected by lung hypoplasia, indicating that a much greater ventilation/perfusion mismatch occurred in DH kittens. However, as the increase in PBF after birth is vital to supply left ventricular preload and maintain cardiac output, maintaining left ventricular output at birth is possibly more important for CDH infants than matching pulmonary perfusion with ventilation.
